# PDLCs and EPCs Co-Cultured on Ta Discs: A Golden Fleece for “Compromised” Osseointegration

**DOI:** 10.3390/ijms22094486

**Published:** 2021-04-26

**Authors:** Hitesh Chopra, Yuanyuan Han, Cheng F. Zhang, Edmond H. N. Pow

**Affiliations:** 1 Division of Restorative Dental Sciences, Faculty of Dentistry, The University of Hong Kong, Hong Kong S.A.R., China; h.chopra@usask.ca (H.C.); zhangcf@hku.hk (C.F.Z.); 2 College of Dentistry, University of Saskatchewan, Saskatoon, SK S7N 5E4, Canada; 3 Division of Applied Oral Sciences and Community Dental Care, Faculty of Dentistry, The University of Hong Kong, Hong Kong S.A.R., China; u3006886@connect.hku.hk

**Keywords:** osteogenesis, neovascularization, osseointegration, VEGFR-2, RUNX-2

## Abstract

Material research in tissue engineering forms a vital link between basic cell research and animal research. Periodontal ligament cells (PDLCs, P) from the tooth have an osteogenic effect, whereas endothelial progenitor cells (EPCs, E) assist in neovascularization. In the present study, the interaction of PDLCs and EPCs with Tantalum (Ta, I) discs, either alone (IP or IE group) or in 1:1 (IPE) ratio was explored. Additionally, surface analysis of Ta discs with respect to different types and cycles of sterilization and disinfection procedures was evaluated. It was observed that Ta discs could be used for a maximum of three times, after which the changes in properties of Ta discs were detrimental to cell growth, irrespective of the type of the protocol. Cell-Disc’s analysis revealed that cell proliferation in the IE group at day 6 and day 10 was significantly higher (*p* < 0.05) than other groups. A cell viability assay revealed increased live cells in the IPE group than in the IP or IE group. Similarly, adhesion and penetration of cells in the IPE group were not only higher, but also had an increased thickness of cellular extensions. RT-PCR analysis revealed that on day 8, both osteogenic (ALP, RUNX-2, and BSP) and angiogenic genes (VEGFR-2, CD31) increased significantly in the IPE group as compared to the IP or IE groups (*p* < 0.05). In conclusion, Ta discs promoted cellular proliferation and increased osteogenic and angiogenic activity by augmenting RUNX-2 and VEGFR-2 activity.

## 1. Introduction

Tissue engineering is making great strides in musculoskeletal research. Classically, the triad of tissue engineering includes stem cells, growth factors, and scaffolds [1]. Research on stem cells, particularly the mesenchymal stem cells (MSCs), has been progressing exponentially over the last decade. The primary advantage of using MSCs is their multipotency, which allows them to differentiate into different cell types. MSCs have been broadly classified into embryonic, fetal, and adult stem cells [2] based on the sources from which they can be isolated and expanded. Periodontal ligaments stem cells (PDLSCs) are the type of MSCs with clonogenicity, self-renewability, and the ability to differentiate into osteoblasts, adipocytes, and chondrocytes [3]. On the other hand, endothelial progenitor cells (EPCs) are unique in forming blood vessels and contributing to neoangiogenesis [4].

In tissue engineering, studying the interactions between stem cells and implant materials is pertinent before embarking on translational research. The use of metallic implants such as Titanium (Ti) and tantalum (Ta) has been increasing gradually over the past decade for medical and dental applications. In dentistry and orthopedics, Ti and its alloys are the most commonly used materials because of their biocompatibility and excellent mechanical properties [5]. Additionally, in orthopedics, trabecular forms of Ta implants have been exploited over the past two decades. Perhaps the best advantage that porous Ta can have is its bone ingrowth because of its trabecular nature, which has been demonstrated in preclinical studies [6,7] and retrieval studies [8,9]. In dentistry, Ta implants have been introduced relatively recently, and therefore, they have a vast potential in clinically compromised conditions such as irradiated bone.

These implant materials are usually used in the form of either disc [10,11,12] or cylinders [13,14] to determine the alteration in cell behavior by exploring various parameters, such as type of metal [15], surface roughness [16], and sterilization techniques [17,18]. Conversely, the source of stem cells can also influence cell response. Rabbit is the most commonly used animal in musculoskeletal research [19], such as in implant studies evaluating osseointegration [13,20,21,22,23]. However, studies investigating the interaction between rabbit PDLCs (rPDLCs) and rabbit EPCs (rEPCs) on porous tantalum trabecular metal (PTTM) implants are lacking. 

Therefore, in the present study, the effects of Ta discs on rPDLCs and rEPCs by culturing cells individually and co-culturing were analyzed in vitro. We hypothesize that Ta discs’ trabecular nature can promote osteogenesis and neoangiogenesis, which signifies the importance of this study before it can be exploited and translated into an animal model for evaluating osseointegration in compromised cases, such as after irradiation therapy or diabetes mellitus.

## 2. Results

### 2.1. Disc Preparation and Evaluation

**Figure 1:**
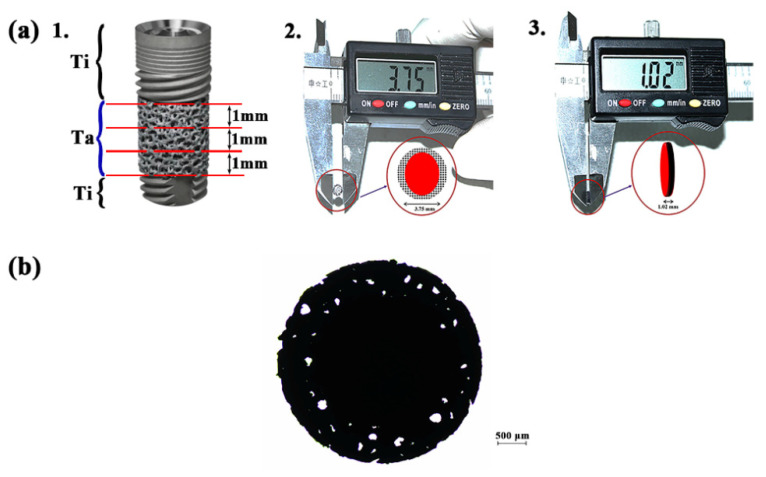
Porous tantalum trabecular metal (PTTM) implant. (**a**) 1. The Ta from the center of the PTTM implant was sectioned into three slices of 1 mm each. 2. The diameter of the Ta Disc. 3. The size of the Ta disc as measured by a vernier caliper. (**b**) Ta disc under the polarizing microscope shows holes where light can pass through, representing interconnectivity.

Ta discs with a diameter of 3.75 mm and a mean width of 1.02 mm (S.D. ± 0.01) were obtained (Figure 1a) and evaluated for various parameters.

#### 2.1.1. Surface Porosity and Surface Texture

Under the microscope, the discs revealed an opaque inner core (black) and outer porous periphery (bright white spots of varying shapes interspersed with the black opaque solid field) (Figure 1b). 

**Figure 2:**
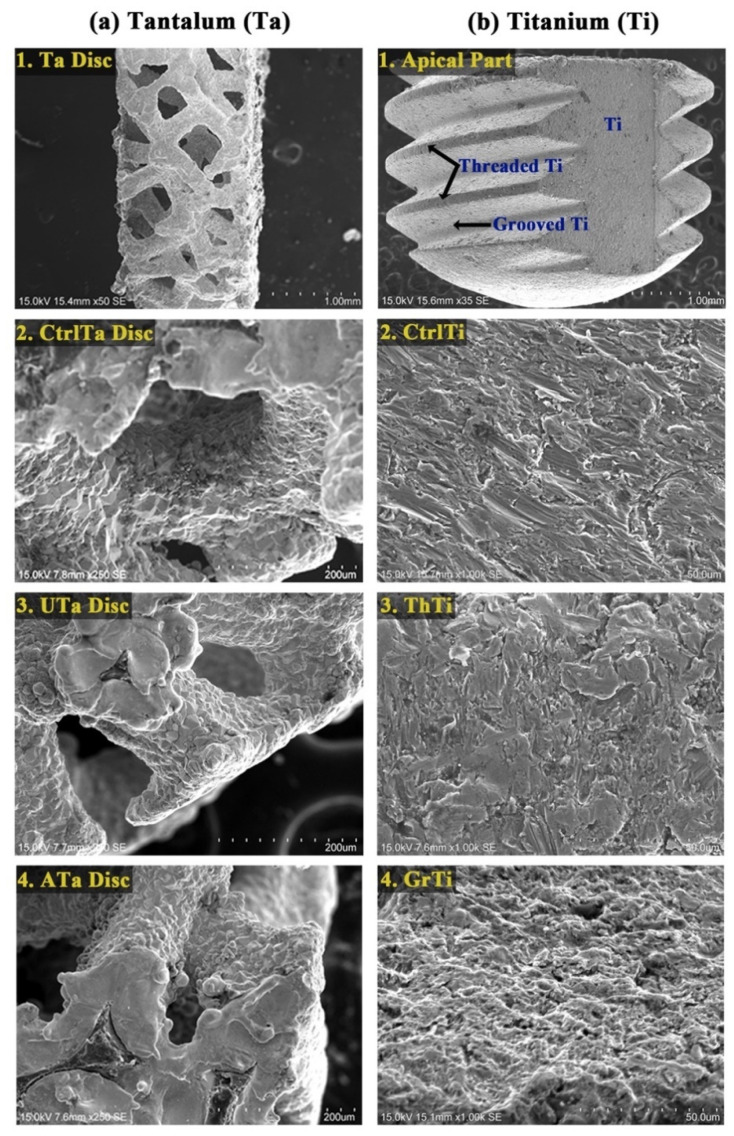
Scanning electron microscopic (SEM) analysis of PTTM implant. (**a**) Ta; 1. Ta discs (Scale bar = 1.00 mm), 2. CtrlTa Disc (Scale bar = 200 µm, 3. UTa Disc (Scale bar = 200 µm, 4. ATa Disc (Scale bar = 200 µm); (**b**) Ti; 1. Apical part of Ti with different surfaces (Scale bar = 1.00 mm), 2. CtrlTi (Scale bar = 50 µm), 3. ThTi (Scale bar = 50 µm), 4. GrTi (Scale bar = 50 µm).

Qualitative analysis of SEM analysis revealed that ultrasonic disinfection and autoclaving of the Ta discs did not significantly change the microstructure of implant discs (Figure 2). However, in the Ti group, GrTi appeared to have a rougher surface compared to the other two groups.

#### 2.1.2. Surface Chemistry

The surface chemistry of all Ta discs was similar (*p* > 0.05) (Figure S1). Oxygen concentration in the autoclaved samples was higher than in the control or ultrasonic samples (*p* < 0.05). On the other hand, the Ti group invariably had the same chemical composition in all types of surfaces (Figure S1).

#### 2.1.3. Surface Roughness

**Figure 3:**
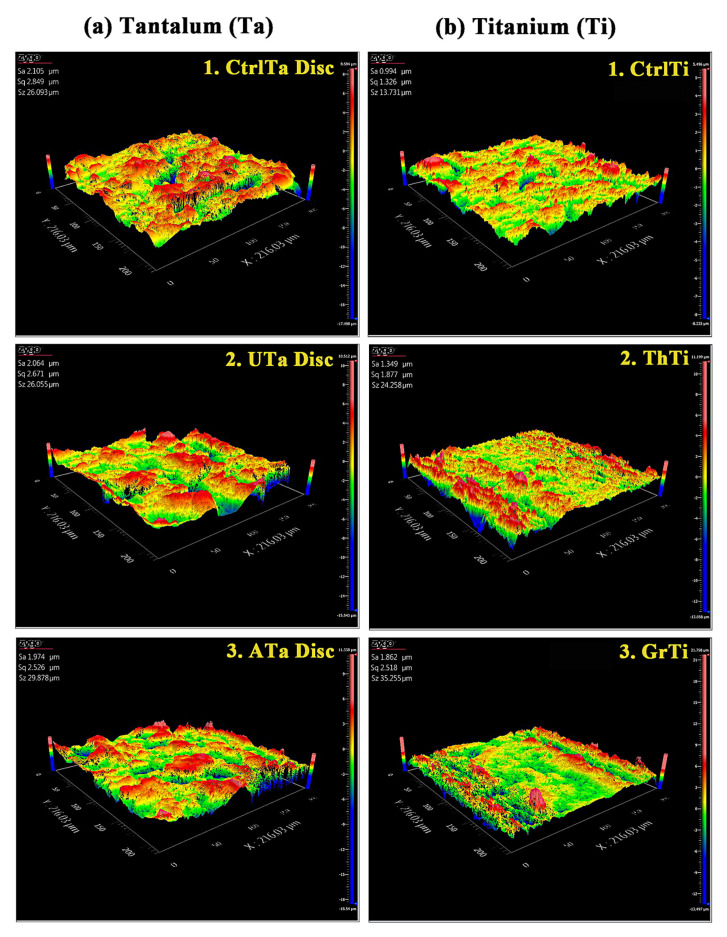
Quantitative analysis of surface roughness (Sa) by an optical interferometer. (**a**) Ta discs; 1. CtrlTa Disc, 2. UTa Disc, 3. ATa Disc; (**b**) Ti; 1. CtrlTi, 2. ThTi, 3. GrTi.

A significant reduction (*p* < 0.05) in the surface roughness (Sa) of ATa discs was observed as compared to the CtrlTa discs. However, the Sa in the ATa group was still higher than in the Ti groups (*p* < 0.05). In the Ti group, Sa of GrTi was significantly higher as compared to CtrlTi or ThTi groups (*p* < 0.05) (Table S1, Figure 3). 

### 2.2. Cell Isolation and Culture

**Figure 4:**
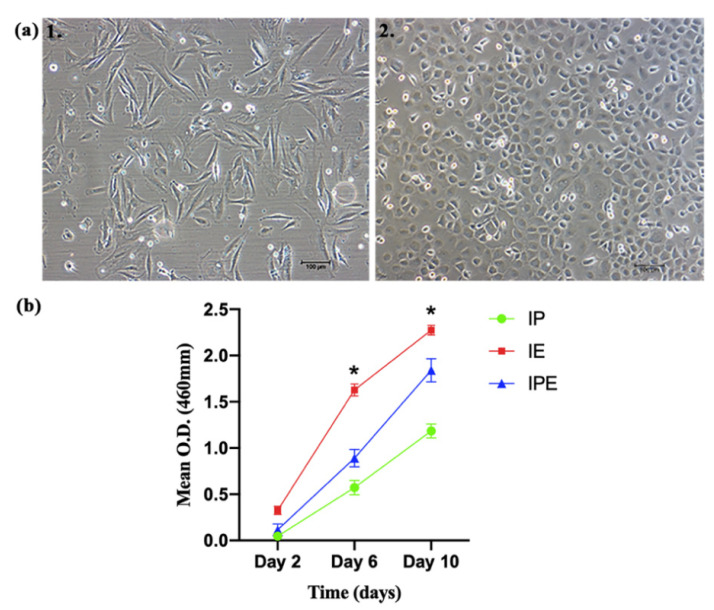
Cells and Cell-Discs (**a**) Morphology of cells at 10×; 1. rPDLCs, 2. rCD34^+^CD133^+^EPCs; (**b**) Growth curve of Cell-Discs between IP, IE, and IPE group (* *p* < 0.05).

Rabbit PDLCs and CD34^+^CD133^+^EPCs were successfully isolated and cultured. The PDLCs had a characteristic fibroblastic morphology, whereas the EPCs had a distinctive cobblestone appearance (Figure 4a).

### 2.3. Cell-Disc Preparation and Evaluation 

Cell-Discs from all three groups were successfully obtained.

#### 2.3.1. Cell Proliferation and Viability

The growth curve (Table S2, Figure 4b) revealed significant differences in cell growth among the groups over time (*p* < 0.05). On day 2, significantly more cell growth was found in the IE group than in the IPE and IP groups (*p* < 0.05). On Day 6 and Day 10, significant differences in cell growth were noticed among all the groups (IE > IPE > IP, *p* < 0.05).

Cell-Discs in all groups demonstrated appropriate cell viability after culturing the cells for eight days (Figure S2). 

#### 2.3.2. Cell Adhesion and Penetration 

**Figure 5:**
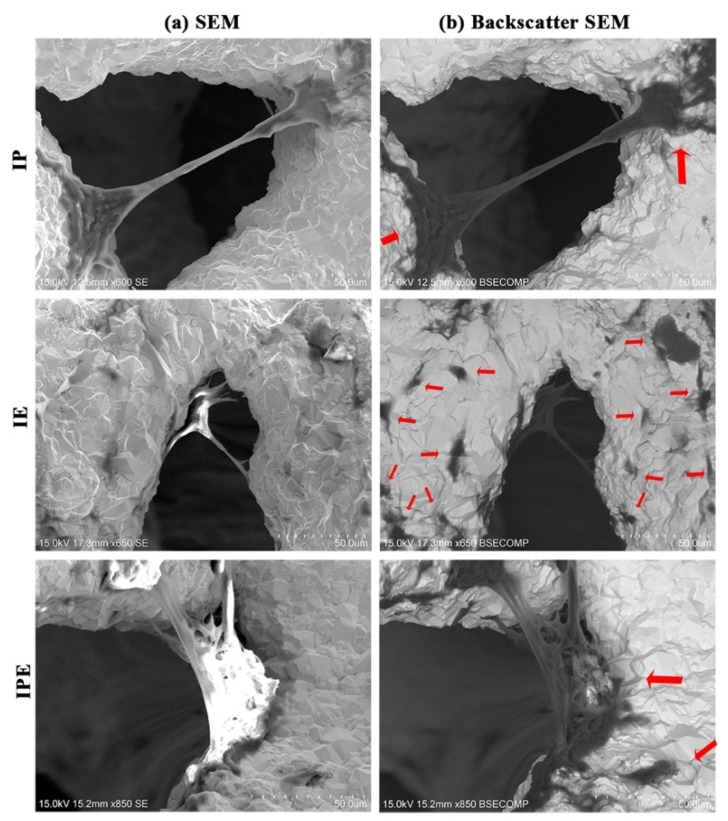
Cell adhesion and cell penetration (**a**) Cell adhesion in Cell-Discs with different IP, IE, and IPE groups under SEM (**b**) Cell penetration (Black spots marked by red arrows) in Cell-Discs with different IP, IE, and IPE groups under backscatter SEM.

SEM revealed cell adhesion in all groups of Ta discs. Single-cell extensions were prominent in the IP group, while multiple cell extensions were evident in the IE group. In the IPE group, thicker cell extensions were noticed (Figure 5a)

Backscatter SEM revealed that both PDLCs and EPCs adhered to the implant surface. However, in the IP group, increased fibroblastic extensions connecting two diametrically opposite ends of a pore were found, whereas, in the IE group, increased surface attachments (black spots) were found. In the IPE group, there was an increased thickness of both extensions and surface attachments (black spots) (Figure 5b).

#### 2.3.3. RT-qPCR for the Expression of Osteogenic and Angiogenic Genes

**Figure 6:**
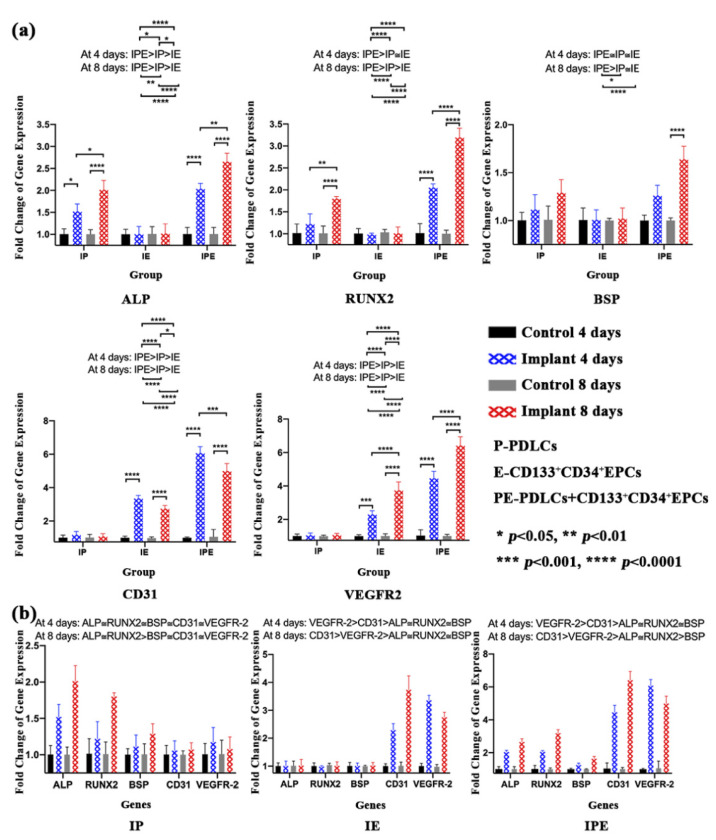
qRT-PCR for Cell-Disc complex in IP, IE, and IPE groups after 4 days and 8 days. (**a**) Individual gene analysis. Expression of osteogenic (ALP, RUNX-2, and BSP) and angiogenic (CD31 and VEGFR-2) genes in IP, IE, and IPE groups at day 4 and day 8. (**b**) Comparative gene analysis. Expression and comparison of osteogenic and angiogenic genes in the IP, IE, or IPE groups at day 4 and day 8.

The RT-qPCR analysis for osteogenic gene analysis revealed that ALP and RUNX-2 genes were significantly higher (*p* < 0.05) at both day 4 and day 8 in the IPE group as compared to other groups (Figure 6a). However, BSP was only significantly higher (*p* < 0.05) on day 8 in the IPE group than in the IE or IP groups. Similarly, angiogenic gene analysis revealed that both CD34 and VEGFR-2 were significantly higher at day 4 and day 8 in the IPE group than in other groups (Figure 6a).

Furthermore, a comparison between osteogenic and angiogenic gene profiles (Figure 6b) revealed no statistically significant differences amongst them at day 4 in the IP group. However, on day 8, both ALP and RUNX-2 were significantly higher (*p* < 0.05) than all other genes (Figure 6b). In the IE group on day 4 and day 8, angiogenic genes were significantly higher than osteogenic genes, with the expression of VEGFR-2 highest at day 4 (*p* < 0.05), while the expression of CD31 peaked at day 8 (*p* < 0.05). In the IPE group, on day 4 and day 8, angiogenic genes were significantly higher than osteogenic genes. Furthermore, there was a shift in osteogenic gene expression from day 4 to day 8. On day 4, there was no significant difference found among ALP, RUNX-2, and BSP, but on day 8, ALP and RUNX-2 were found to be significantly higher than BSP (*p* < 0.05) (Figure 6b).

## 3. Discussion

This is the third study in the sequence of four studies where the ultimate goal was to evaluate the osseointegration in an irradiated rabbit model by a tissue engineering approach. In the first two studies, PDLCs and EPCs were isolated, characterized, and compared with corresponding human cells [24,25], whereas the present in vitro study investigated the cellular behavior of rPDLCs and rEPCs on Ta discs, serving an axiomatic link between basic cell research and in vivo animal studies. Since our ultimate aim was to enhance osseointegration using tissue-engineered implants in irradiated bone where both osteogenesis and angiogenesis are compromised, co-culturing two distinct cell populations in an optimized ratio might have been a better alternative than using single cells. In addition, studies have shown that PDLCs have better osteogenic potential than other dental-derived stem cells [26]. On the other hand, EPCs were selected because they contribute directly to blood vessel formation and not indirectly through a paracrine mechanism, such as in dental stem cell lines or other stem cells [4]. Additionally, the synergistic relationship of PDLCs and EPCs was evaluated because co-culture can augment the osteogenic [27] or angiogenic potential, which is critical for irradiated bone with limited blood supply and bone cells.

Furthermore, as surface characteristics can influence cell behavior, we evaluated the surface texture, surface chemistry, and Sa of Ta discs. To evaluate the cell response over the scaffold-like metal discs/cube, these in vitro cell studies required many discs/cubes. However, PTTM, unlike other implant materials, is an expensive substrate. Therefore, the first objective in the implant evaluation was to determine whether Ta discs could be reused again after sterilization and disinfection procedures. We tested the discs after four repeated cycles of culturing, followed by sterilization (autoclave) or disinfection (ultrasonic), followed by re-culturing. Qualitative analysis by SEM exhibited no change in surface texture, whereas quantitative analysis revealed that Ta discs surface roughness decreased more significantly than the CtrlTa group. The high pressure and temperature might be one of the possible reasons for the flattening of surface grains in the autoclaved group. However, ultrasonic treatment did not influence the surface roughness when compared to the control group. The surface compositions were also altered, with oxygen level increasing gradually from the ultrasonic group to autoclaved discs. A similar study also found that the commercially pure Ti (CpTi) surfaces contained more oxygen, carbon, and nitrogen after sterilization [18]. Additionally, steam autoclaving was reported to have detrimental effects on cell attachment [18]. However, in the present study, we found no significant differences in cell attachment and proliferation with Ta discs for the first three cycles. The most plausible explanation is the porous nature of Ta discs, which could be maintained for three cycles. After three cycles, the gradual accumulation of cell debris and flattening of grains resulted in decreased cell attachment and proliferation.

It is imperative to note that Ti was used to compare against Ta, as Ti is the most common material for dental implants, and to the best of our knowledge, no data are available for comparing the surface roughness of Ti and Ta. It is well-known that surface roughness alters cell adhesion and proliferation. Therefore, if Sa of Ta discs is found to be no different from Ti, it completely defies the purpose of culturing cells over Ta. We also investigated the effect of cleansing techniques on Ta discs because Ta is approximately 25 times more costly than Ti of the same size. The data were useful for our latter experiments if Ta discs could be reused. In the present study, Ta discs without any cleansing procedure were the control group (CtrlTa), while Ti discs were not the control, but they gave the readers an overview and some baseline data of how Ti was different from Ta, qualitatively and quantitatively.

In the cell proliferation experiment, it was found that all the cells proliferated on Ta discs, but to a variable extent. The IE group had significantly higher growth at all time points as compared to the IP or IPE group. One possible reason might be that a homogenous CD133^+^CD34^+^EPCs population was used in the IE group, while in the other 2 groups, heterogeneous PDLCs were used instead. Furthermore, it was observed that cell proliferation of rPDLCs and rEPCs on PTTM were similar to the in vitro studies described earlier [24,25]. 

SEM is a vital tool in material studies, which can reveal changes in surface texture, different stages of cell attachment, as well as any possible interactions between both. The earliest study described four stages in cell attachment and spreading: attachment of cells at the point of contact with the substratum, centrifugal growth of filopodia, cytoplasmic webbing, and flattening of the central mass [28]. In the present study, we also observed all the four stages in cell attachment and proliferation. However, branched cells with multiple cytoplasmic extensions and flattened cells were visibly more pronounced in the IE group as compared to the IP group. Furthermore, elongated cytoplasmic extensions, giving a characteristic spindle-shape morphology, were more distinct in the IP group. In the IPE group, all four stages, as well as augmentation of cell attachment by increased branching, thickening of cytoplasmic extensions, or increased surface area of flattened cells, were evident. 

To study the interaction between dental implant surfaces, such as CpTi or PTTM, and cells, various investigators have used different types of cell lines, such as osteogenic cells [29], HUVEC [30], EPCs [31], PDLSCs [32], DPSCs [11], and BMSSCs [33]. In the present study, we used PDLCs (IP), EPCs (IE), as well as co-cultured PDLCs and EPCs in a 1:1 ratio (IPE) because ALP, BSP, and RUNX-2 genes were shown to be expressed significantly higher (*p* < 0.05) in a 1:1 ratio (PDLSCs:HUVEC) as compared to monocultures or other ratios of 2:1 or 1:5 [27]. Our results were synchronous with the corresponding study [27] where the expression of BSP and RUNX-2 were higher on day 8 as compared to day 4. In contrast, the ALP gene expression was also higher on day 8 as compared to day 4. The change in ALP activity might be due to the time required for the adaptation of cells to the Ta discs. The above studies [11,27,29,30,31,32,33] evaluated only osteogenic or angiogenic expression, whereas in the present study, we analyzed individual as well as the synergistic relationship between osteogenic and angiogenic activity.

Despite limited resources and some limitations, such as the absence of rabbit-specific markers for immunofluorescence analysis, technical problems while evaluating adhesion quantitatively by SEM on cell cultured porous Ta discs, and the use of 2D culture techniques for culturing a 3D material like a Ta disc, the present study demonstrated rationally that Ta discs’ trabecular nature can promote osteogenesis and neoangiogenesis. The use of these tissue-engineered implants can have immense potential in dentistry and orthopedics by opening new avenues in treatment modalities, such as “personalized implants” where the body’s own cells can be cultured over the implant for a “personalized therapy”.

## 4. Materials and Methods

### 4.1. Disc Preparation

PTTM dental implants (Zimmer, Parsippany, NJ, USA) were cut cross-sectionally into discs of 1 mm thick with a molybdenum wire EDM (Electrical Discharge Machining) (Figure 1a). The size of the cut discs was verified using a digital caliper (RS Components Ltd., Northampton shire, UK). Additionally, for comparison of surface characteristics only, the solid apical Ti part of the implant was also used.

### 4.2. Disc Evaluation

The Ta discs were divided into three groups: group A: control (Ctrl Ti), group B: disinfection by ultrasonic cleaning in 70% ethanol (UTa), and group c: sterilization by autoclave (ATa). The changes in surface characteristics were evaluated according to the surface porosity, surface texture, chemical composition, and roughness. Additionally, the apical portion of Ti was earmarked into three different groups: group A: control Ti (Ctrl Ti), group B: Ti present on the threads (Threaded Ti, ThTi), and group C: Ti present in between two threads (Grooved Ti, GrTi) for observation and intragroup comparison of the surface characteristics of Ti similar to Ta discs.

#### 4.2.1. Surface Porosity

The sectioned Ta discs were examined under a polarizing light microscope (Nikon Eclipse LV 100 POL, Nikon Corporation, Tokyo, Japan) for evaluating surface porosity, and images were captured by a digital camera (Nikon DS-Ri1, Tokyo, Japan). The images were then analyzed using NIS elements AR 3.1 imaging software (Nikon Corporation, Tokyo, Japan).

#### 4.2.2. Surface Texture

The effect of disinfection or autoclaving on the specimens’ surface microtopography was evaluated by a scanning electron microscope (SEM; SU1510, Hitachi, Tokyo, Japan). The discs were examined at 250× magnification. Similarly, Ti groups were also evaluated under the same magnification. 

#### 4.2.3. Surface Chemistry

To identify the specimen’s chemical composition, energy-dispersive X-ray analysis was performed for all the groups of Ta discs and Ti using energy-dispersive X-ray spectroscopy (EDS detector; SDD3310, IXRF, Austin, TX, USA). The results were then analyzed by EDS software, Iridium Ultra (IXRF, Austin, TX, USA).

#### 4.2.4. Surface Roughness 

The surface roughness (Sa) in all the groups of porous Ta discs and Ti was evaluated by a Zygo laser interferometric non-contact profiler system (Nexview, Zygo, Middlefield, CT, USA).

### 4.3. Cell Isolation and Culturing

#### 4.3.1. Rabbit PDLCs

rPDLCs were isolated and characterized as described in the previous study [24]. A complete culture medium (all from Gibco, Life sciences; Thermo Fisher Scientific, Waltham, MA, USA) was used for culturing rPDLCs at 37 °C and 5% CO_2_ in a humidified chamber. The media was changed two times per week. After 70% to 80% confluence was reached, trypsin-EDTA (0.25%) was used to detach the adherent cells. Only cells from P4-P6 were used in the current study.

#### 4.3.2. Rabbit CD34^+^CD133^+^EPCs

rCD34^+^CD133^+^EPCs were isolated and characterized as described in the previous study [25]. EGM-2MV (Lonza, Basel, Switzerland) supplemented with 10% fetal bovine serum (FBS) was used to culture rEPCs at 37 °C and 5% CO_2_ in a humidified chamber. The media was changed every two days. Trypsin-EDTA (0.25%) was used to detach cells after the culture was 70–80% confluent. Only cells from P4–P6 were used in the current study.

### 4.4. Cell-Disc Complex (Cell-Disc) Preparation

rPDLCs and rCD34^+^CD133^+^EPCs were cultured with the discs either alone or in a 1:1 ratio. In brief, three groups were made: group 1: implant PDLCs (IP), group 2: implant EPCs (IE), and group 3: implant+PDLCs+EPCs (IPE). α-MEM was used for PDLCs, EGM-MV was used for EPCs, and α-MEM: EGM-2MV (1:1) was used for co-culturing PDLCs:EPCs (1:1). After detaching the adherent cells, the cell suspension was adjusted to 1 × 10^4^ cells/µL. 

A total of 1000 cells/discs were used for the growth curve, while 2 × 10^4^ cells were used for the rest of the experiments. Appropriate cell suspension of 20 µL was seeded over the discs in a 96-well plate and incubated for 4 hr. After incubation, the discs were carefully transferred to other wells. The wells were then supplemented with 200 µL of the medium (EM-2MV or MEM, or EGM-2MV: α-MEM), and the medium was changed every two days. Cell-Discs were evaluated variously for cell proliferation, cell viability, cell adhesion, cell penetration, and gene expression.

### 4.5. Cell-Disc Evaluation

#### 4.5.1. Cell Proliferation

The proliferative potential of rPDLCs and rCD34^+^CD133^+^EPCs in various groups was determined by the CCK-8 assay (Sigma-Aldrich, St. Louis, MO, USA). In brief, all groups at the P5 passage were seeded on the discs according to the protocol described above. The growth was measured every two days for 10 days. The medium was replaced every two days. On the day of evaluation, the medium was replaced by a phenol red-free culture medium containing 1% P/S (medium) and 10μL of CCK-8 (working medium). After incubating plates for 2 hr at 37 °C, the discs were carefully removed from each well. The plates were then measured for absorbance at 460 nm (O.D., optical density) by a microplate reader. The experiment was performed in triplicate. The negative control consisted of the working medium and 10μL of the CCK-8 solution. 

#### 4.5.2. Cell Viability

Cell viability was measured using the live/dead^®^ viability/cytotoxicity kit for mammalian cells (L3224, Thermo Fisher Scientific, Waltham, MA, USA). Briefly, for each well, a working solution of 0.25 mL PBS, 0.5 µL of Ethidium homodimer (EthD-1, 4 µM), and 0.125 µL Calcein-AM (2 µM) was prepared. After culturing Cell-Discs in each group for 8 days, the medium was replaced with a working solution and incubated for 45 min, followed by examination under a fluorescent microscope. The experiment was performed in triplicate. Calcein AM produced green fluorescence in live cells (ex/em ~495 nm/~515 nm), whereas EthD-1 generated bright red fluorescence in dead cells (ex/em ~495 nm/~635 nm). 

#### 4.5.3. Cell Adhesion

Cell adhesion to discs for each group was assessed by a scanning electron microscope (SEM, SU1510, Hitachi, Tokyo, Japan). Briefly, after culturing the Cell-Discs for 8 days, these Cell-Discs were carefully transferred to a 6-well plate and dried overnight in a dehumidifier, followed by examination under SEM. The experiments were repeated a minimum of three times.

#### 4.5.4. Cell Penetration

Cell penetration in each group was assessed using the backscatter SEM (BSSEM, SU1510, Hitachi, Tokyo, Japan). Briefly, after analysis of Cell-Discs from each group with SEM, these Cell-Discs were further analyzed by BSSEM for a demonstration of cell growth within the implant.

#### 4.5.5. Real-Time Quantitative Reverse-Transcription Polymerase Chain Reaction (RT-qPCR) for the Expression of Osteogenic and Angiogenic Genes

Expression of alkaline phosphatase (ALP), runt-related transcription factor 2 (RUNX-2), bone sialoprotein (BSP), vascular endothelial growth factor 2 (VEGFR-2), platelet endothelial cell adhesion molecule (PECAM-1), and CD31 genes were detected in the I, IP, and IPE groups at 4 and 8 days by RT-qPCR, using primer pairs designed using Primer 3 software (Table S3). Briefly, 5 discs were used for culturing the PDLCs (IP), CD133^+^CD34^+^EPCs (IE), and PDLCs+CD133^+^CD34^+^EPCs (IPE) group, whereas 96-well plates were used as a negative control for each group. The cells were then pooled and homogenized in a TRIzol reagent at days 4 and 8 to extract total RNA. The SuperScript First-Strand Synthesis System for RT-PCR (Invitrogen) was used to synthesize cDNA. After evaluation, cDNA was amplified by SYBR Green-based RT-qPCR in a PCR machine (ABI Prism 7300 Sequence Detection System, Applied Biosystems, Thermo Fisher Scientific, Waltham, MA, USA). The housekeeping gene, glyceraldehyde 3-phosphate dehydrogenase (GAPDH), was used to perform data normalization, whereas comparisons were made by the ∆∆CT method.

### 4.6. Statistical Analysis

A one-way ANOVA was used to analyze the difference in the mean surface roughness between different groups. For the growth curve and analysis of gene expression, a 2-way repeated-measures ANOVA was applied for testing the difference in mean growth and mean ∆∆CT, respectively, between the three groups (IP, IE, and IPE group) at the same time point and between different time points within the same group. The pairwise comparisons were adjusted by Bonferroni adjustment. The above tests were performed as the two-sided tests at the 0.05 significance level, using IBM SPSS Statistics 25 (IBM Corporation, Armonk, NY, USA).

## 5. Conclusions

rPDLCs and rEPCs can be successfully cultured over Ta discs. Furthermore, Ta is a bio-compatible surface that demonstrated cell viability, cell adhesion, and proliferation when either PDLCs or EPCs were cultured alone or co-cultured. Additionally, Cell-Discs co-cultured with rPDLCs and rCD34^+^CD133^+^EPCs demonstrated enhanced osteogenic and angiogenic activity.

## Data Availability

All data generated or analyzed during this study are included in this publication, supported with a supplementary file.

## Supplementary Materials

The following are available online at https://www.mdpi.com/article/10.3390/ijms22094486/s1: Figure S1: Energy-dispersive X-ray spectroscopy (EDX) of various samples. (**a**) Ta discs; 1. CtrlTa Disc, 2. UTa Disc, 3. ATa Disc (**b**) Apical Ti; 1. CtrlTi, 2. ThTi, 3. GrTi. Figure S2: Live/dead staining of Cell-Discs in different IP, IE, and IPE groups on day eight. Table S1: Primer sequences for RT-qPCR of osteogenic and angiogenic genes. Table S2: Optical Interferometer. One-way repeated measures ANOVA for comparing surface roughness (Sa) in different samples. Table S3: Growth Curve of PDLCs and EPCs, either cultured alone (IP/IE) or both (IPE) on Ta discs.
