# Supplementary material for: PDLCs and EPCs Co-Cultured on Ta Discs: A Golden Fleece for “Compromised” Osseointegration
Source: Int J Mol Sci. 2021 Apr 26;22(9):4486. doi: 10.3390/ijms22094486 (PMC8123461; doi:10.3390/ijms22094486)
Supplement: Supplementary file 1 [file ijms-22-04486-s001.zip › ijms-1167287-supplementary.pdf]

International Journal of *Molecular Sciences*

MDPI

Supplemental Material

PDLCs and EPCs Co-cultured on Ta Discs: A Golden Fleece for "Compromised" Osseointegration

Hitesh Chopra [1,2], Yuanyuan Han [3], Cheng F. Zhang [1], Edmond H.N. Pow [1,*]

[1] Division of Restorative Dental Sciences, Faculty of Dentistry, The University of Hong Kong, Hong Kong S.A.R., China; h.chopra@usask.ca (H.C.); zhangcf@hku.hk (C.F.Z.)
[2] College of Dentistry, University of Saskatchewan, Saskatoon, SK S7N 5E4, Canada;
[3] Division of Applied Oral Sciences and Community Dental Care, Faculty of Dentistry, The University of Hong Kong, Hong Kong S.A.R., China; u3006886@connect.hku.hk
* Correspondence: ehnpow@hku.hk; Tel.: +852-2859-0309

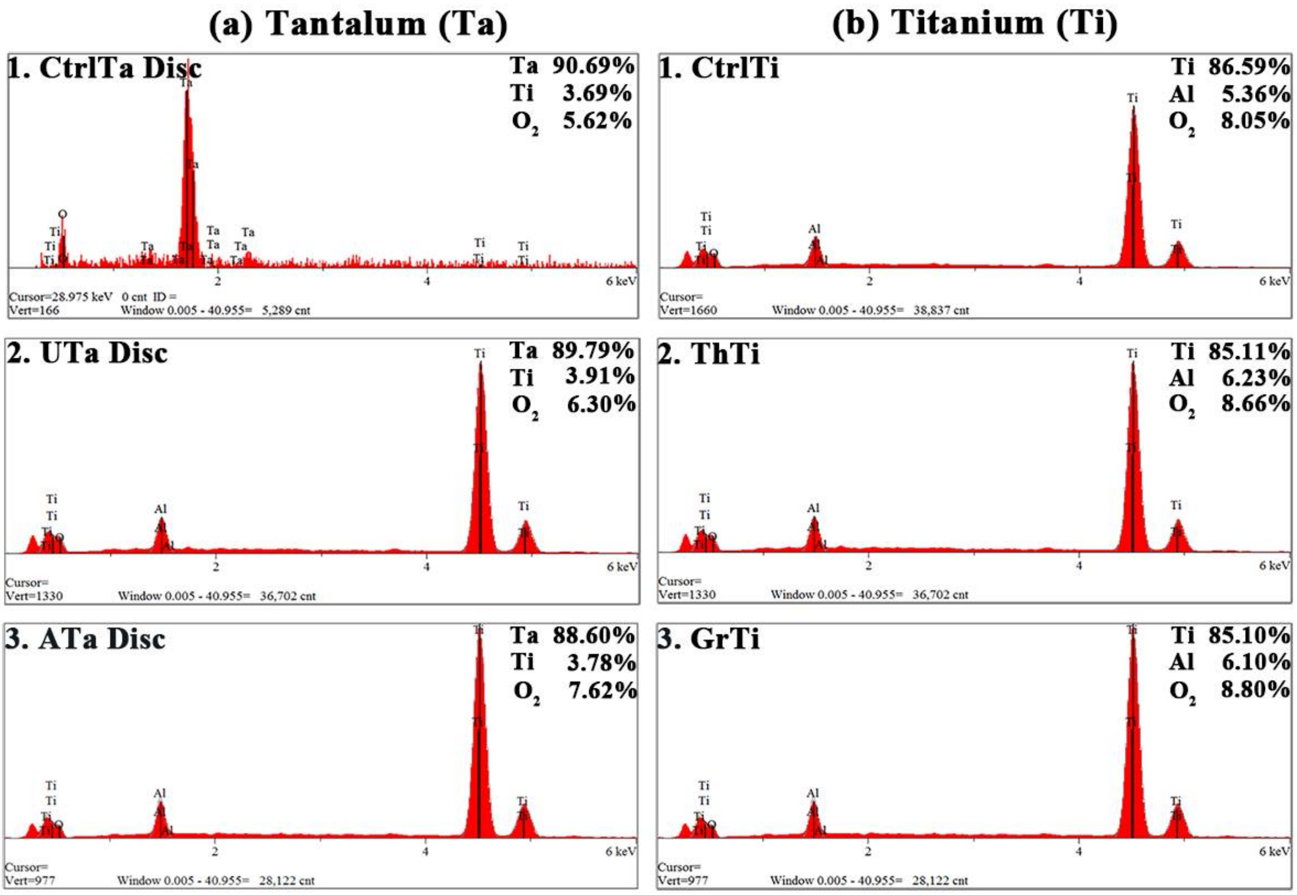

Figure S1. Energy-dispersive X-ray spectroscopy (EDX) of various samples. **(a)** Ta discs 1) CtrlTa Disc 2) UTa Disc 3) ATa Disc **(b)** Apical Ti 1) CtrlTi 2) ThTi 3) GrTi.

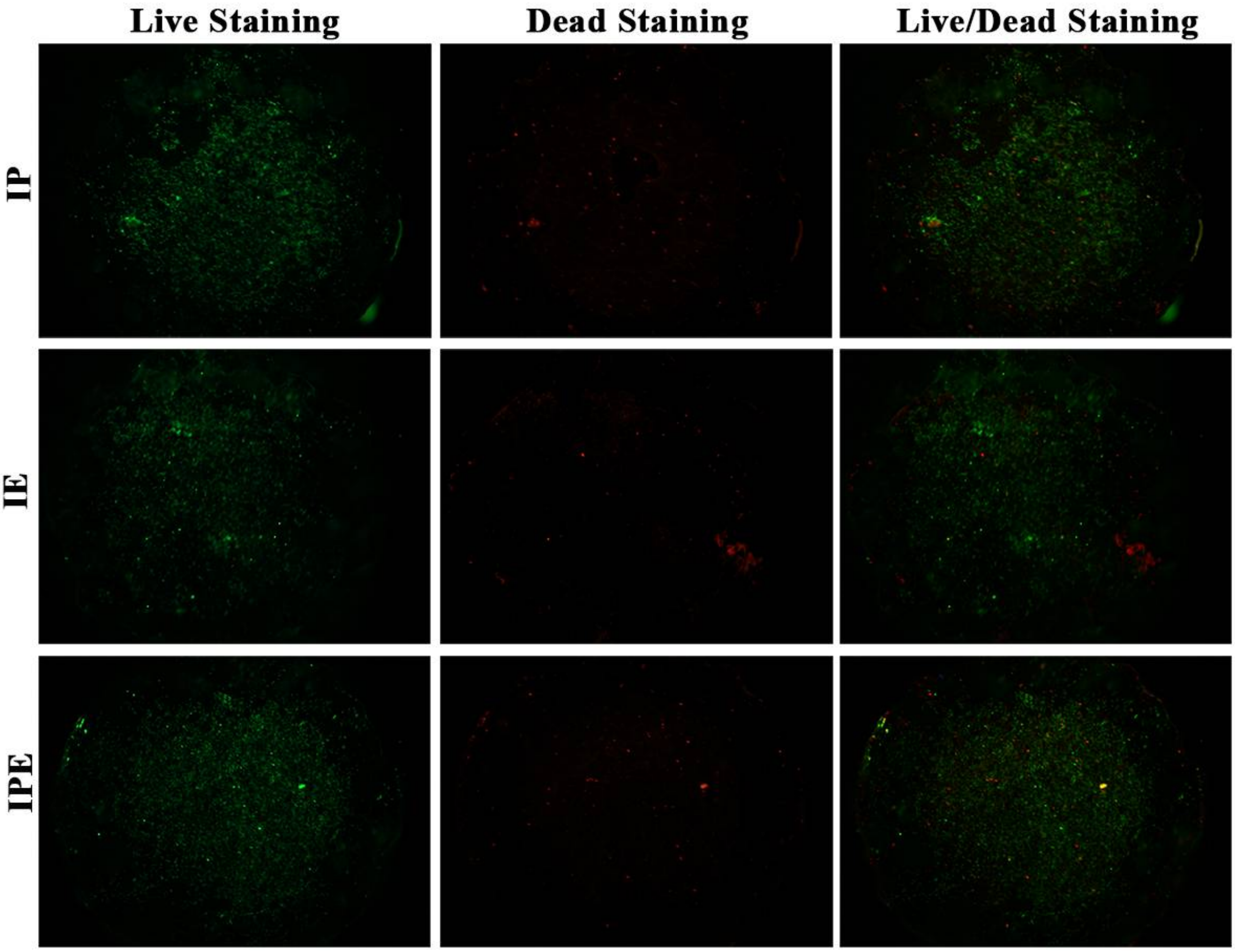

Figure S2. Live/dead staining of Cell-Discs in different IP, IE, and IPE groups on day 8.

Table S1. Optical Interferometer. One-way repeated measures ANOVA for comparing surface roughness (Sa) in different samples.

Groups	Mean ± Std. Deviation (N=9)	p-value	*Multiple comparisons
1 (PTi)	.98±.022	< 0.05	4>6>3>2>1
2 (ThTi)	1.33±.065		5=4
3 (GrTi)	1.86±.042		5=6
4 (Ta)	2.11±.007		
5 (UTa)	2.06±.021		
6 (Ata)	1.98±.015		

Post-Hoc Tukey HSD test for multiple comparisons with adjustment (statistically significant at $p < 0.05$).

Table S2. Growth Curve of PDLCs and EPCs either cultured alone (IP/IE) or both (IPE) on Ta discs.

x	Groups[a]	Mean ± Standard deviation (N=3)	Pairwise comparison			
			Between groups	Within group[b]		
			p value	rIP	rIE	rIPE
Day2	1	0.047±0.022	2>3=1	Day 2 <	Day 2 <	Day 2 <
	2	0.327±0.044	($p < 0.05$)	Day 6 <	Day 6 <	Day 6 <
	3	0.116±0.063		Day 10	Day 10	Day 10
Day6	1	0.572±0.078	2>3>1			
	2	1.628±0.065	($p < 0.05$)			
	3	0.891±0.095				
Day10	1	1.185±0.075	2>3>1			
	2	2.273±0.053	($p < 0.05$)			
	3	1.841±0.126				

a. Group 1: rIP; Group 2: rIE; Group 3: rIPE; b. Post-Hoc Tukey HSD test for multiple comparisons with adjustment (statistically significant at $p < 0.05$).

Table S3. Primer sequences for RT-qPCR of osteogenic and angiogenic genes.

Genes	Accession number	Primer	Primer Sequence (5'-3')	Amplicon length (bp)	T m (°C)
**GAPDH**	NM_001082253.1	F	TGGTGAAGGTCGGAGTGAAC	121	62
		R	ATGTAGTGGAGGTCAATGAATGG		
**ALP**	XM_017346489.1	F	AGAAACCCTTCACTGCCATC	128	60
		R	GGTAGTTGTTGTGAGCGTAGTC		
**RUNX-2**	XM_008262992.2	F	CGCATTCCTCATCCCAGTAT	118	60
		R	GCCTGGGGTCTGTAATCTGA		
**BSP**	XM_008267617.2	F	CTACCGTTCCGGAGTGATTT	95	62
		R	TCAGTGGCCCTCATTTACAG		
**VEGFR-2**	NM_001195670.1	F	GAAGAGGAAGTGTGCGACCC	167	60
		R	TCCGACTGGTTGTCATCTGG		
**CD31**	XM_008271716.2	F	CAAGGAGAACGGGGACAGAC	225	60
		R	ATCACCCCGATGACAACCAC		
